# Sexual intercourse frequency and fecundability in the Norwegian mother, father and child cohort study

**DOI:** 10.1371/journal.pone.0357286

**Published:** 2026-09-23

**Authors:** Kristina W. Dreiås, Liv G. Kvalvik, Mari L. Warp, Siri E. Håberg, Cecilia H. Ramlau-Hansen, Hans Ivar Hanevik

**Affiliations:** 1 Fertility department, Telemark Hospital, Porsgrunn, Norway; 2 Centre for Fertility and Health, Norwegian Institute of Public Health, Oslo, Norway; 3 Department of Global Public Health and Primary Care, University of Bergen, Bergen, Norway; 4 Department of Gynecology and Obstetrics, Vestfold Hospital, Tønsberg, Norway; 5 Department of Public Health, Aarhus University, Aarhus, Denmark; Ordu University, TÜRKIYE

## Abstract

**Objective:**

The aim of this study was to explore the association between frequency of intercourse in the four weeks leading up to conception and fecundability among women trying to conceive in a pregnancy cohort.

**Methods:**

The Norwegian Mother, Father and Child Cohort Study is a pregnancy cohort in Norway with recruitment between 1999 and 2008. This study includes self-reported questionnaire data from 64 193 women with planned, naturally conceived pregnancies.

Mean time to pregnancy for women trying to conceive was calculated for different intercourse frequencies and the Jonckheere-Terpstra test for trend was performed for time to pregnancy across ordered groups of intercourse frequency. The cycle specific probability of conception (fecundability ratio) was calculated for intercourse frequencies of less than once, 1–2, 3–4, > 4 times per week through proportional probability regression with adjustment for the women’s age and education level. Several supplementary analyses were performed to account for additional covariates such as BMI, parity, endometriosis and irregular menstrual cycles.

**Results:**

The mean time to pregnancy decreased with intercourse frequency from 4.8 months for less than once per week to 3.7 months for > 4 times weekly, however the decrease was primarily evident for the group reporting intercourse > 4 times weekly compared to lower intercourse frequencies. There was a significant trend in TTP across ordered intercourse frequency groups (p < 0.0001), indicating progressively shorter TTP with higher intercourse frequencies. We found a 16% higher chance of conceiving in any cycle (fecundability ratio 1.16 (95% CI:1.14–1.19)) among women reporting intercourse >4 times per week compared to the reference group (1–2 times per week) within the analytic cohort. This association was consistent in supplementary analyses accounting for BMI, parity, menstrual cycle regularity and endometriosis. There were no clear differences in fecundability between intercourse frequencies of less than once, 1–2 and 3–4 times per week.

**Conclusion:**

Among women with planned pregnancies that resulted in natural conception, intercourse >4 times per week in the four weeks leading up to conception, was associated with a higher cycle probability of conceiving, whereas differences across lower frequency categories were limited.

## Introduction

Sexual intercourse during the periovulatory phase of the menstrual cycle is fundamental for natural conception. The frequency of sexual intercourse varies widely between couples trying to conceive and is associated with several demographic characteristics such as age and educational level [1–5]. A reciprocal biological influence between sexual activity and fecundability is also at play, where normal hormonal function is associated with both timing and frequency of sexual activity [6–8].

In recent years, studies indicate a downward trend in intercourse frequency in several populations [4,9–13]. The implications of this trend for fecundability and fertility remains uncertain for several reasons.

Only a few small-scale studies [14–16] have examined the association between intercourse frequency and fecundability. These found that a higher intercourse frequency was associated with higher fecundability. No large-scale epidemiological studies have been conducted to confirm their findings. Furthermore, time-to-pregnancy (TTP), a common measure of fecundability, rarely accounts for intercourse frequency and could become influenced by menstrual cycles without periovulatory intercourse [14]. Another controversial implication of intercourse frequency is whether frequent ejaculations could reduce sperm concentration, potentially affecting chances of pregnancy [17].

In this study, we used a large population-based cohort to examine if intercourse frequency was associated with fecundability and time to pregnancy.

## Materials and methods

### Study design and population

The Norwegian Mother, Father and Child Cohort Study (MoBa) is a pregnancy cohort study conducted by the Norwegian Institute of Public Health [18]. Pregnant women and their partners were recruited at 15–18 weeks of pregnancy prior to their routine ultrasound appointment between 1999 and 2008 from most hospitals in Norway with a labor and delivery ward. The participation rate was 41%. The cohort consists of approximately 114 000 children, 95 000 mothers and 75 000 fathers. The MoBa data collection was performed under approval from The Regional Committees for Medical and Health Research Ethics and is currently regulated by the Norwegian Health Registry Act. Written consent was obtained from all participants upon recruitment. The current study was approved by The Regional Committees for Medical and Health Research Ethics of South/East Norway, project number 277291.

The data for the current study was extracted from the first pregnancy questionnaire at inclusion in MoBa and data from the Medical Birth Registry (MBRN). All data were fully anonymized and were accessed April 10, 2025.

**Fig 1:**
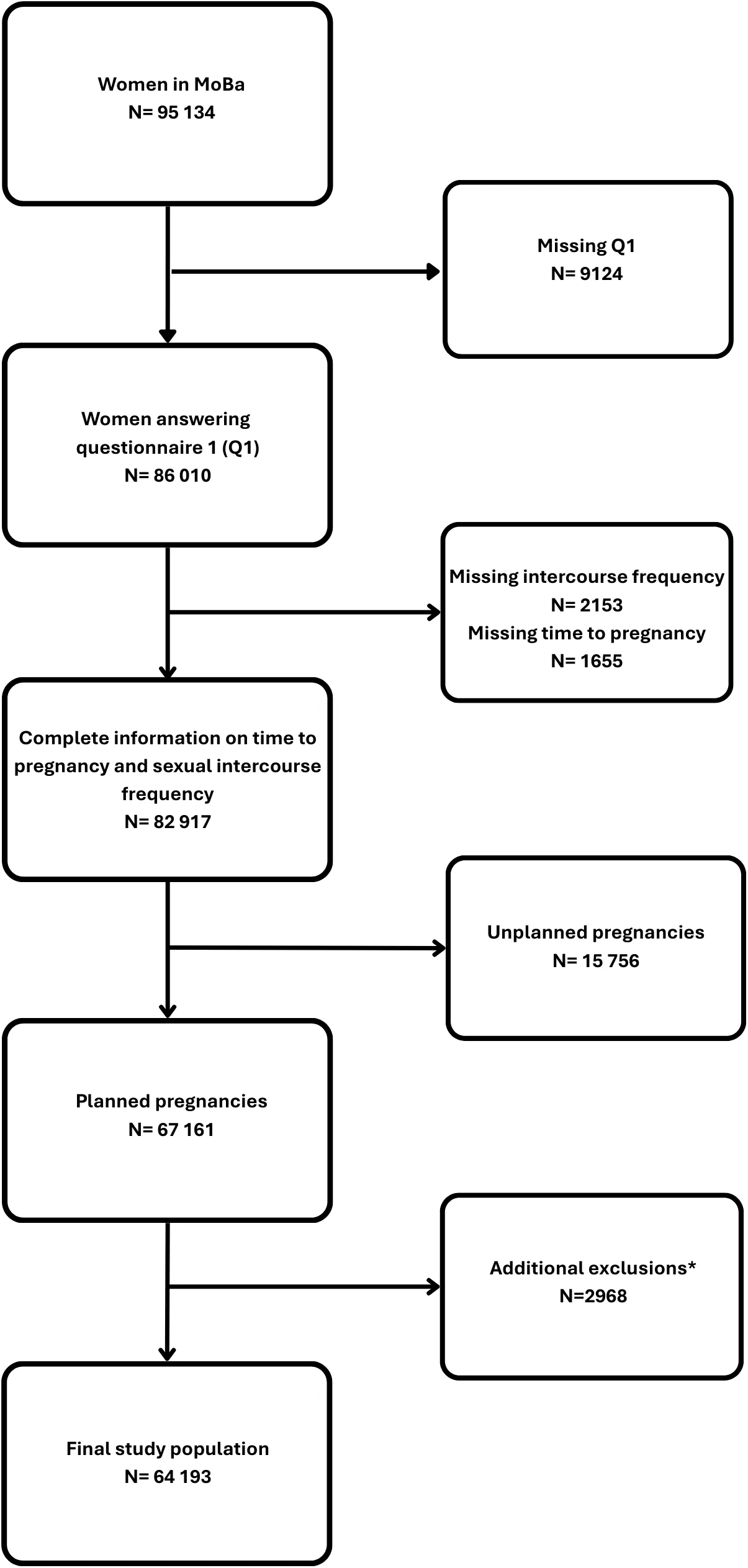
Flowchart of inclusions and exclusions in study population. MoBa: The Norwegian Mother, Father and Child Cohort Study. *Participants answering “Never” on the question about intercourse frequency prior to pregnancy (N = 292), pregnancies resulting from assisted reproductive technologies (N = 2219), time-to-pregnancy >36 months (N = 1321).

To avoid that women participated more than once in the study population, only the first pregnancy in MoBa was included among women participating with several pregnancies. Women with missing information on intercourse frequency or time to pregnancy were excluded from the study, as were those pregnancies resulting from assisted reproductive technology (ART). Women reporting time to pregnancy above 36 months (99^th^ percentile) were excluded due to risk of bias [19]. Women with unplanned pregnancies were excluded since TTP is reported on the premise that the pregnancy was planned. S1 Table shows the characteristics of planners compared to the excluded non-planners. Fig 1 describes the inclusions and exclusions resulting in the final study population of 64 193 women.

### Data analysis

The study’s main exposure variable was intercourse frequency in the four weeks leading up to conception, and the main outcomes were TTP and fecundability ratios based on TTP.

Intercourse frequency was assessed based on the question “How often did you have sexual intercourse during the four weeks before you became pregnant?” and the reply options were: “Every day”, “5-6 times a week”, “3-4 times a week”, “1-2 times a week”, “1-2 times every two weeks”, “Less than 1-2 times every 2 weeks” and “Never”.

Women were categorized into 4 groups of intercourse frequency: more than 4 times a week, 3–4 times a week, 1–2 times a week and less than once a week. Women replying “Never” or more than one option were excluded from further analysis.

TTP was assessed from the questions “Was this pregnancy planned?” and “If yes, how many months did you have regular intercourse without contraception before you became pregnant?” where the reply options were “Less than 1 month”, “1-2 months” or “3 months or more”. Those replying “3 months or more”, were further asked to report the number of months. To construct a discrete TTP variable based on these responses, certain assumptions were necessary. We assumed that the women who reported “Less than 1 month”, conceived during the first menstrual cycle of attempting pregnancy, while those who reported “1-2 months”, conceived during the second cycle. We therefore assigned those answering “Less than 1 month” to TTP = 1 month and those reporting “1-2 months” to TTP = 2 months. Those who answered “3 months or more” without further specifying the number of months, were assigned TTP = 3 months, and the remaining women were assigned the number of reported months. Women who reported use of a contraceptive method when becoming pregnant were reclassified as non-planners and excluded from the study population. We also excluded women who used contraception in the past 12 months while reporting a TTP of more than 12 months. Where cycle length was available (97.2%), TTP was modeled in corrected cycles by dividing TTP by cycle length in days and multiplying by 28. In the remaining women (2.8%), TTP was left unchanged with months as the unit.

Potential confounding factors were identified through literature searches. Female age and educational level were recognized as likely confounding factors [1–5,20,21]. Female age at childbirth was extracted from the MBRN whilst highest ongoing or completed education were extracted from the recruitment questionnaire.

Body mass index (BMI), parity, endometriosis and irregular menstrual cycles were also considered as possible confounders. BMI, parity and irregular menstrual cycles were not included in the main analysis because existing literature does not demonstrate a clear association with intercourse frequency [1–5]. Endometriosis is known to be associated with both female sexual dysfunction and fecundability [22,23], but in our study population, information on endometriosis prevalence was limited to self-reported diagnosis (N = 678), which we considered insufficient for inclusion in the main analysis. However, these additional characteristics were included in supplementary analyses.

Fecundability ratio (FR), a risk ratio that expresses the ratio of per-cycle probability of conception in an exposed group relative to a reference group [24], was calculated for each group of intercourse frequency using proportional probability regression on all complete cases with censoring at 12 months. To facilitate this analysis, the TTP data was formatted into a binary outcome variable for each cycle up to the 12^th^ cycle indicating whether conception occurred in that specific cycle or not. Clustering was applied to correlate the cycles of each woman. The reference was defined as an intercourse frequency of 1–2 times weekly. We adjusted for female age and educational level in the main analysis with several supplementary analyses to explore the possible impact of BMI, parity, endometriosis and irregular menstrual cycles. We also performed the Jonckheere-Terpstra test, a non-parametric rank-based test for trend [25], to determine whether there was a statistically significant ordered trend in TTP across groups of intercourse frequency. Stata version MP19.5 was used for all statistical analyses.

## Results

### Participant characteristics

A total of 64 193 participants were included in the study. Study participant characteristics by intercourse frequency are presented in Table 1. The majority of participants reported an intercourse frequency of 1–2 (35.5%) or 3–4 (37.3%) times per week. The mean age at childbirth was 30.1 years, approximately half (53%) were nulliparous. The majority (67%) had attained or were pursuing higher education. Women who reported frequent intercourse (>4 times weekly), were more often in the younger age groups, nulliparous and had a lower education level. The excluded non-planners were in general younger, with less attained education and had a higher prevalence of irregular menstrual cycles.

**Table 1:** Characteristics of study population by categories of intercourse frequency.

	Total	<1	1-2	3-4	>4
**Total, n (%)**	64193 (100)	9037 (14.1)	22792 (35.5)	23913 (37.3)	8451 (13.2)
**Age, n (%)**					
<25	6055 (9.4)	453 (5.0)	1530 (6.7)	2482 (10.4)	1590 (18.8)
25-29	22876 (35.6)	2441 (27.0)	7846 (34.4)	9297 (38.9)	3292 (39.0)
30-34	25108 (39.1)	4072 (45.1)	9568 (42.0)	8975 (37.5)	2493 (29.5)
>34	10154 (15.8)	2071 (22.9)	3848 (16.9)	3159 (13.2)	1076 (12.7)
Missing	0	0	0	0	0
Mean	30.1				
**Parity, n (%)**					
0	33964 (52.9)	3595 (39.8)	11270 (49.5)	13815 (57.8)	5284 (62.5)
1	20495 (31.9)	3918 (43.4)	7829 (34.4)	6712 (28.1)	2036 (24.1)
2	8228 (12.8)	1339 (14.8)	3166 (13.9)	2854 (11.9)	869 (10.3)
≥3	1506 (2.4)	185 (2.1)	527 (2.3)	532 (2.2)	262 (3.1)
Missing	0	0	0	0	0
**Education level, n (%)**					
<High school	4068 (6.3)	520 (5.8)	1268 (5.6)	1371 (5.7)	909 (10.8)
High school	16561 (25.8)	2153 (23.8)	5564 (24.4)	6167 (25.8)	2677 (31.7)
University/college ≤ 4 years	26637 (41.5)	3845 (42.6)	9753 (43.8)	10066 (42.1)	2973 (35.2)
University/college > 4 years	15587 (24.3)	2311 (25.6)	5750 (25.2)	5815 (24.3)	1711 (20.3)
Missing	1340 (2.1)	208 (2.3)	457 (2.0)	494 (2.1)	181 (2.1)
**Diagnosis of endometriosis** ^**a**^ **, n (%)**					
Yes	678 (1.1)	103 (1.1)	248 (1.1)	246 (1.0)	81 (1.0)
No	63515 (98.9)	8934(98.9)	22544 (98.9)	23667 (99.0)	8370 (99.0)
Missing	0	0	0	0	0
**Menstrual cycle regularity**^**b**^, n (%)					
Regular	48308 (75.5)	6887 (76.2)	17246 (75.7)	17924 (75.0)	6251 (74.0)
Irregular	15719 (24.6)	2125 (23.5)	5486 (24.1)	5934 (24.8)	2174 (25.7)
Missing	166 (0.3)	26 (0.3)	55 (0.2)	60 (0.3)	25 (0.3
**Body mass index, n (%)**					
<18.5	1815 (2.9)	239 (2.6)	621 (2.7)	665 (2.8)	290 (3.4)
18.5-24.9	41092 (65.9)	5621 (62.2)	14439 (63.4)	15652 (65.5)	5380 (63.7)
25.0-29.9	13748 (22.0)	2041 (22.6)	5059 (22.2)	4900 (20.5)	1748 (20.7)
≥30.0	5712 (9.2)	848 (9.4)	2035 (8.9)	2038 (8.5)	791 (9.4)
Missing	1826 (2.8)	288 (3.2)	638 (2.8)	658 (2.8)	242 (2.9)
Mean	24.0				

(%) are column percentages.

^a^Self-reported.

^b^Regular defined as menstruation at regular intervals of 21–35 days.

### Association between intercourse frequency, time to pregnancy and fecundability

**Fig 2:**
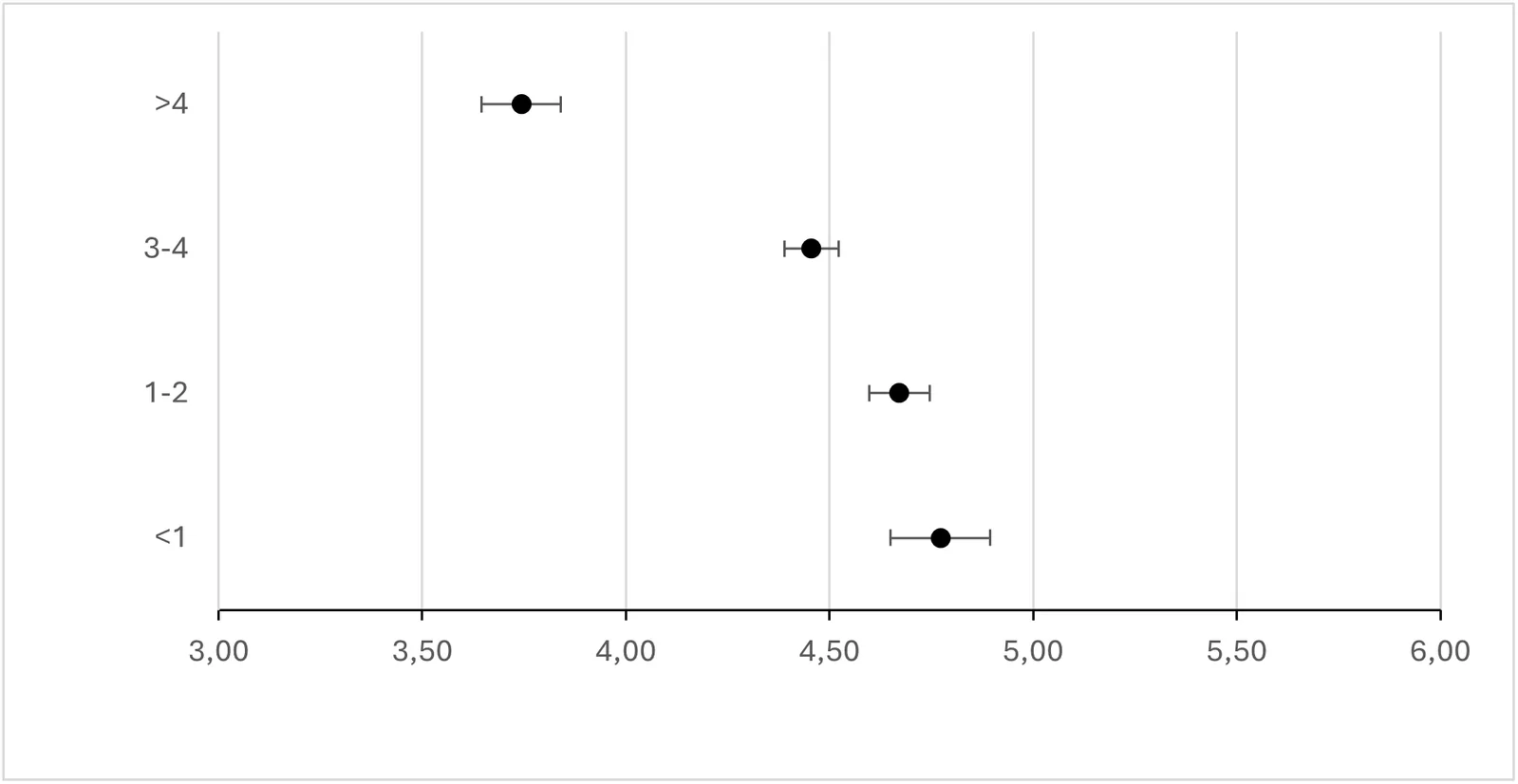
Mean TTP by categories of sexual intercourse frequency. X-axis: Mean TTP in months (95% CI). Y-axis: Sexual intercourse frequency (times per week).

The mean TTP was 4.5 months, with variation according to intercourse frequency ranging between 3.7 months for women with intercourse> 4 times per week and 4.8 for women with intercourse less than once per week, however the main difference was observed for the group reporting intercourse > 4 times per week compared to the groups reporting less frequent intercourse (Fig 2, S2 Table). The Jonckheere-Terpstra test demonstrated a statistically significant trend in TTP across ordered intercourse frequency groups (JT statistic = 3.90x10^7^, z-value = 7.76, two-sided p < 0.0001), indicating progressively shorter TTP with higher intercourse frequencies.

**Fig 3:**
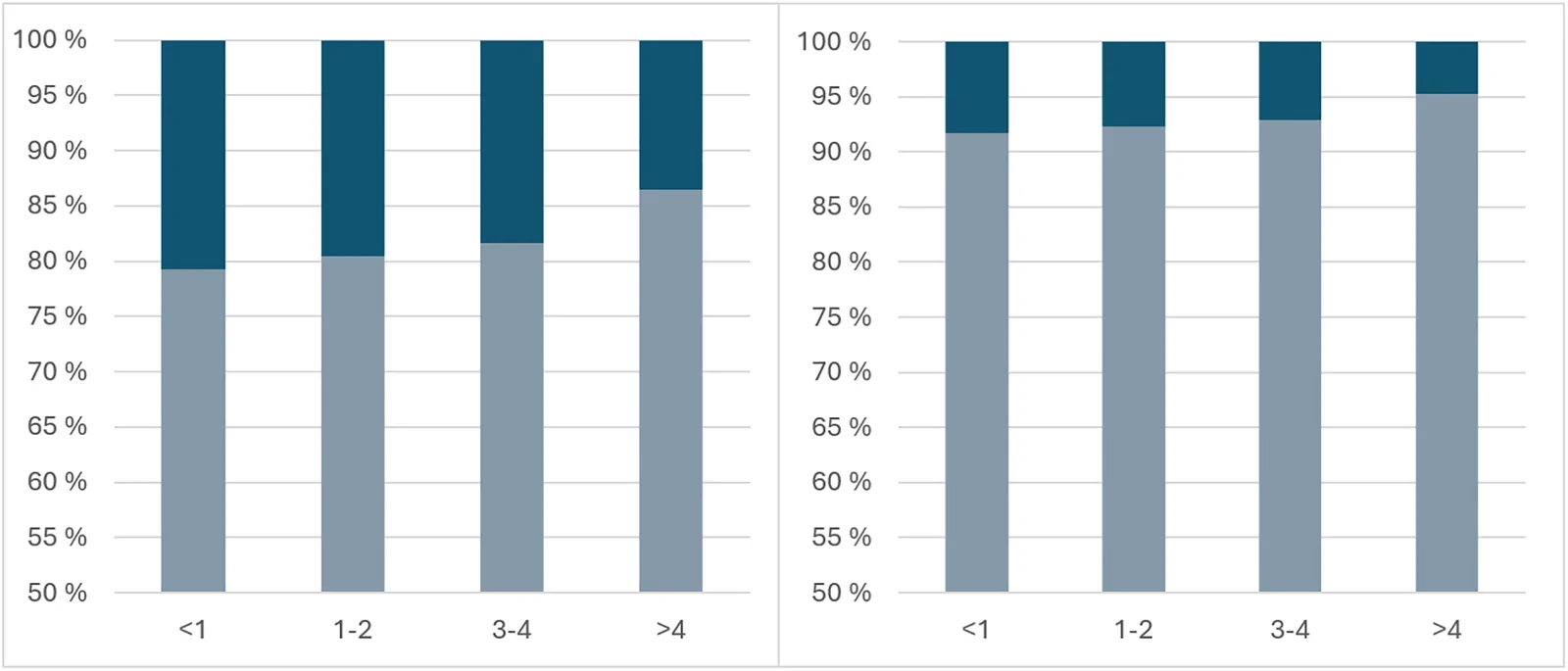
Percentage of study population with a TTP within 6 months and 12 months by categories of sexual intercourse frequency. Left: Percentage of study population with a TTP within 6 months. Right: Percentage of study population with a TTP within 12 months. Light colored proportion of bars represent pregnant, dark colored proportion of bars represent non-pregnant. X-axis: Categories of sexual intercourse (times per week).

In this study of planned pregnancies where all women eventually conceived spontaneously, a total of 82% conceived within 6 months and 93% within 12 months of trying. Higher intercourse frequency (>4 times weekly) was associated with a greater proportion of conceptions within 6 and 12 months with modest differences in the lower frequency groups (Fig 3, S2 Table). The same pattern was observed across all age groups (S1 fig).

**Fig 4:**
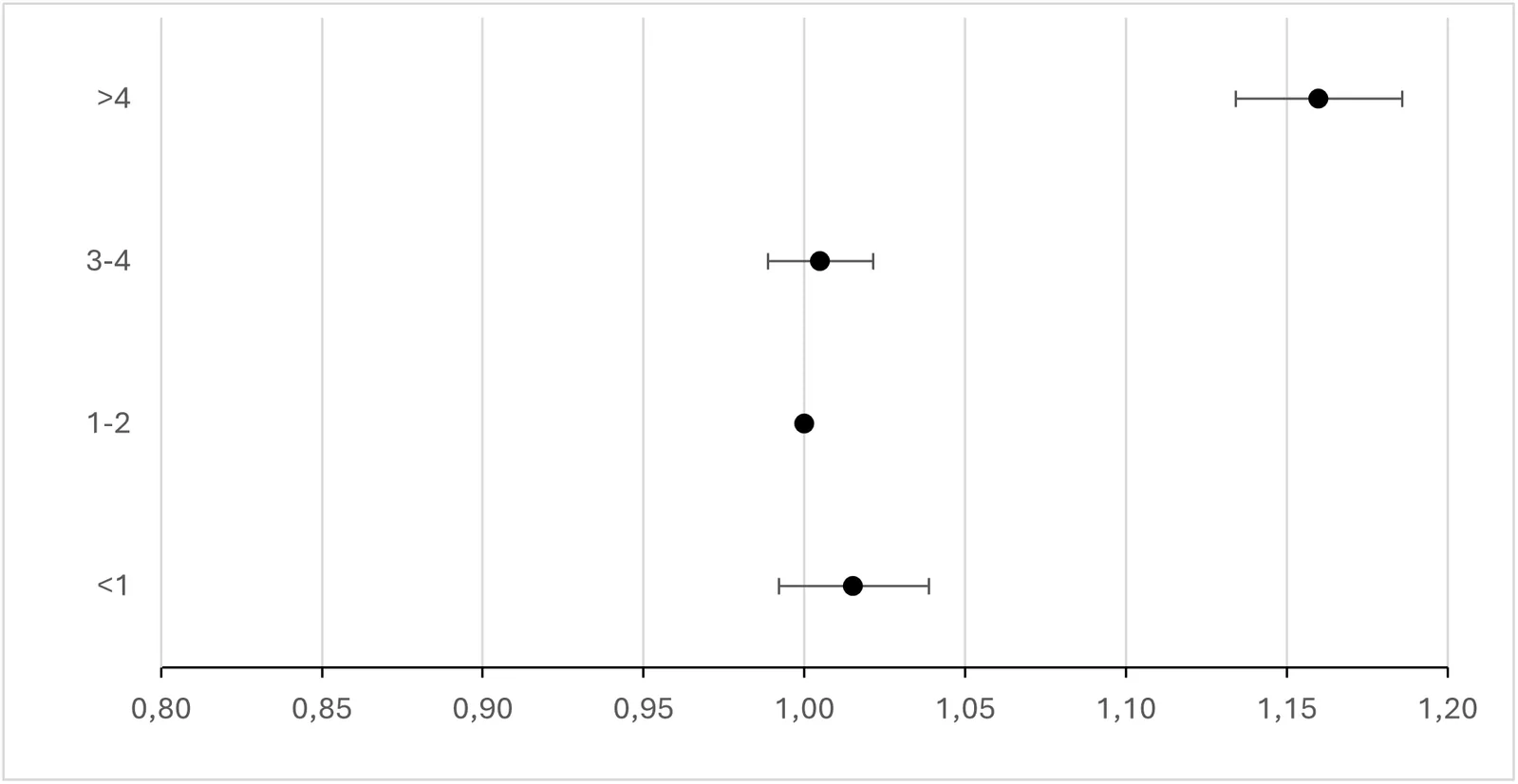
Fecundability ratios by categories of sexual intercourse frequency. Fecundability ratios adjusted for age, completed or ongoing education level with sexual intercourse frequency of 1-2 times per week as the reference group. X-axis: Fecundability ratio (95% CI). Y-axis: Sexual intercourse frequency (times per week).

Fig 4 and Table 2 presents unadjusted and adjusted FRs for categories of intercourse frequency where 1–2 times per week is the reference group. In the adjusted model, women reporting high intercourse frequency (> 4 times weekly) had a 16% increased chance of conceiving in a cycle (FR 1.16 (95% CI:1.14–1.19)) compared to the reference group in the analytic cohort. There were no differences in FR when comparing an intercourse frequency of less than once, 1–2 and 3–4 times per week.

**Table 2:** Fecundability ratio (FR) by categories of intercourse frequency.

Intercourse frequency (times per week)	Participants, n (%)	FR, crude (95% CI)	FR, adjusted^a^ (95% CI)
>4	8270 (13.2)	1.18 (1.16-1.21)	1.16 (1.14-1.19)
3-4	23419 (37.3)	1.02 (1.01-1.04)	1.01 (0.99-1.02)
1-2	22335 (35.5)	1 (reference)	1 (reference)
<1	8829 (14.1)	1.00 (0.97-1.02)	1.01 (0.99-1.04)
Total	62853		

^a^Adjusted for female age and education level.

### Supplementary analyses for additional covariates

The association between an intercourse frequency of >4 times per week and increased fecundability was consistent across stratified analyses by parity and BMI, in sensitivity analyses excluding women with irregular menstrual cycles and women with endometriosis as well as in a second adjustment model including BMI, parity and menstrual cycle regularity as additional covariates (S3-S7 Tables).

When stratifying by parity, a stronger association between intercourse frequency > 4 times per week and fecundability (FR 1.23) was observed among nulliparous women. In this group, a slightly higher FR was also seen with an intercourse frequency of 3–4 times/week compared to the reference group (FR 1.03 (95% CI: 1.01–1.05)), whereas an intercourse frequency of less than once per week was associated with a slightly lower fecundability (FR 0.97 (95% CI: 0.93–1.00)) (S3 Table). The association between intercourse frequency and fecundability did not differ substantially across strata of BMI (<18.5, 18.5–24.9, 25.0–29.9, ≥ 30), with higher fecundability ratios observed among women reporting intercourse > 4 times weekly in all BMI groups (S4 Table). In the sensitivity analysis excluding women with irregular menstrual cycles, we observed a slightly higher adjusted FR (FR1.03 (95% CI: 1.01–1.04)) for women reporting an intercourse frequency of 3–4 times/week compared to the reference group (S5 Table). Exclusion of women with endometriosis did not materially change the results, however, these findings should be interpreted with caution due to the limited quality of the endometriosis data in this study (S6 Table). The second adjustment model showed similar results to those of the main model (S7 Table).

## Discussion

In this pregnancy cohort, including more than 64 000 women with planned, naturally conceived pregnancies, intercourse more than 4 times weekly in the four weeks leading up to conception was associated with a higher cycle probability of conceiving and a shorter time to pregnancy. This association between intercourse frequency and fecundability was consistent across supplementary analyses exploring potentially modifying effects of BMI, parity, menstrual cycle regularity and endometriosis.

Few and small studies have previously analyzed the association between intercourse frequency and fecundability with findings that were in line with our results.

A cohort study with longitudinally/prospectively collected information on intercourse frequency from Japan among 80 couples trying to conceive found that higher intercourse frequency was associated with higher probability of pregnancy by 24 weeks of follow up (OR 1.23 (95% CI: 1.02–1.47)) [14]. Another study from Spain based on retrospective questionnaire data from 2510 women recruited during their postpartum hospital admission, found the highest fecundability among couples reporting an intercourse frequency of seven to eight times a week [15]. Using mathematical modeling methods to examine different patterns of intercourse and time to pregnancy, Stanford and Dunson [16] demonstrated a non-linear decrease in mean time to pregnancy with increased intercourse frequency.

The association between intercourse frequency and fecundability is often explained by the higher likelihood of intercourse within the fertile days of the menstrual cycle. However, we found no difference in fecundability among women reporting intercourse frequency of 3–4 or less than once per week compared to the reference group in the main analysis, which could be explained by intercourse more precisely timed with ovulation in these groups. For nulliparous women, we found small, but statistically significant differences between intercourse frequency groups of 3–4, 1–2 and less than once per week as well as a stronger association between high intercourse frequency and fecundability, which we speculate could indicate that nulliparous women have less timed intercourse when attempting to conceive. When excluding women with irregular menstrual cycles, we also found a slightly increased FR in women reporting intercourse 3–4 times per week compared to the reference group. Whether this represents a modifying effect of cycle regularity on reproductive behavior or is merely due to the restricted sample is unclear.

An alternative explanatory mechanism could be a reverse causality between intercourse frequency and TTP. Prolonged TTP might contribute to reduced intercourse frequency due to diminished persistence in attempting conception, psychological stress and negative impact on relationship quality [26,27]. We mitigated this potential to some extent by censoring at 12 months in our main analysis.

Furthermore, there are indications that being sexually active is associated with a healthy reproductive function in women, including favorable menstrual cycle characteristics and higher likelihood of ovulation [6,7]. In men, sexual function, fertility and overall health likewise appear to be closely intertwined [28,29]. These observations imply that both men and women with a healthy sexual function and who thereby engage in more frequent intercourse, may inherently be more fecund.

One limitation of the study is the possibility of overestimating fecundability since MoBa participants were all pregnant, as well as the exclusion of pregnancies resulting from ART. Conversely, the exclusion of unplanned pregnancies may have led to the omission of the most fecund participants which differed from the study population in several ways such as age, education level and menstrual cycle characteristics. These restrictions might limit generalizability of the findings to the general population. Another limitation is the retrospective, self-reported nature of the data which introduces a potential for recall bias. Although previous studies with comparable recall periods indicate that retrospectively collected data generally overestimates intercourse frequency, the degree of overestimation seems to remain undifferentiated among subgroups [30–32]. In our study, data on intercourse frequency was only available for the four weeks preceding conception, which raises the concern for temporal proximity bias. Furthermore, we lacked information on the timing of intercourse within the menstrual cycle, a key factor given that the likelihood of conception varies throughout the cycle and increases with well-timed intercourse around ovulation [33]. We also did not have information on the male partner’s sperm quality which could influence fecundability [34]. Despite these limitations, our study has important strengths, including a large number of participants, the possibility to adjust for multiple covariates and robust findings through various supplementary analyses.

Our findings may contribute to the understanding of the complex interplay between biological and behavioral factors that defines female reproductive function. They might also add context for interpreting fecundability studies by highlighting the influence that intercourse frequency may have on TTP. The relevance of intercourse frequency also extends to the definition of infertility, i.e., a disease characterized by the failure to establish a clinical pregnancy after 12 months of regular unprotected sexual intercourse [35], which does not specify the extent of “regular intercourse”. Assessment of intercourse frequency may provide useful context when evaluating infertility, although further studies including non-conceiving couples are warranted. Furthermore, these results support that frequent sexual intercourse, despite the potential for reduced sperm count [17], does not negatively impact fecundability in a fertile population.

## Conclusion

Among women with planned pregnancies that resulted in natural conception, an intercourse frequency of > 4 times per week in the four weeks leading up to conception, was associated with higher fecundability and shorter time to pregnancy. Our findings highlight that frequent intercourse was associated with reduced time to pregnancy in all studied subgroups of age, BMI, parity and menstrual cycle regularity and support that frequent intercourse was not associated with lower estimated fecundability within this cohort of planned, naturally conceived pregnancies. Further studies are warranted to explore the relationship between intercourse frequency and fecundability in greater detail, particularly regarding the timing of intercourse within the menstrual cycle.

## Supporting information

S1 Table Comparison of characteristics of non-planners and planners. ^a^Self-reported. ^b^Regular defined as menstruation at regular intervals of 21–35 days.
(DOCX)

S2 Table Time to pregnancy by categories of intercourse frequency. (DOCX)

S3 Table Fecundability ratio (FR) by categories of intercourse frequency stratified by parity. ^a^Adjusted for age and education level.
(DOCX)

S4 Table Fecundability ratio (FR) by categories of intercourse frequency stratified by body mass index (BMI) group. ^a^Adjusted for age and education level.
(DOCX)

S5 Table Fecundability ratio (FR) by categories of intercourse frequency excluding women with irregular menstrual cycles^a^ (N = 15719). ^a^Defined as not menstruation at regular intervals of 21–35 days. ^b^Adjusted for age and education level.
(DOCX)

S6 Table Fecundability ratio (FR) by categories of intercourse frequency, excluding women with endometriosis^a^ (N = 678). ^a^ Self-reported. ^b^ Adjusted for age and education level.
(DOCX)

S7 Table Fecundability ratio (FR) by categories of intercourse frequency with alternative adjustment model^a^. ^a^Adjusted for age, education level, BMI, parity, menstrual cycle regularity.
(DOCX)

S1 Fig Percentage of pregnancies achieved within 6 months and 12 months by age group and intercourse frequency. The numbers inside the colored squares represent the percentage of women that had a time to pregnancy within 6 months (left side) and 12 months (right side). The column on the right explains the color scale, ranging from the lowest percentage (red) to the highest percentage (dark green).
(TIF)
