# Supplementary material for: Sexual intercourse frequency and fecundability in the Norwegian mother, father and child cohort study
Source: PLoS One. 2026 Sep 23;21(9):e0357286. doi: 10.1371/journal.pone.0357286 (PMC13600493; doi:10.1371/journal.pone.0357286)
Supplement: Supplementary file 1 [file pone.0357286.s001.docx]

**S1 Table. Comparison of characteristics of non-planners and planners.**

|  | Total | Planners | Non-planners |
| --- | --- | --- | --- |
| Intercourse frequency |  |  |  |
| <1 | 13146 (16.2) | 9217 (14.2) | 3929 (24.9) |
| 1-2 | 28525 (35.2) | 23212 (35.6) | 5313 (33.7) |
| 3-4 | 28365 (35.0) | 24194 (37.1) | 4171 (26.4) |
| >4 | 10913 (13.5) | 8535 (13.1) | 2378 (15.1) |
| Total | 80949 | 65158 | 15791 |
| Age, n (%) |  |  |  |
| <25 | 10036 (12.4) | 6124 (9.4) | 3912 (24.8) |
| 25-29 | 28009 (34.6) | 23061 (35.4) | 4948 (31.3) |
| 30-34 | 29836 (36.9) | 25481 (39.1) | 4355 (27.6) |
| >34 | 13068 (16.1) | 10492 (16.1) | 2576 (16.3) |
| Total | 80949 | 65158 | 15791 |
| Missing | 0 |  |  |
| Mean | 29.9 | 30.2 | 28.7 |
| Parity, n (%) |  |  |  |
| 0 | 43536 (53.8) | 34486 (52.9) | 9050 (57.3) |
| 1 | 24120 (29.8) | 20799 (31.9) | 3321 (21.0) |
| 2 | 10839 (13.4) | 8343 (12.8) | 2496 (15.8) |
| ≥3 | 2454 (3.0) | 1530 (2.4) | 924 (5.9) |
| Total | 80949 | 65158 | 15791 |
| Missing | 0 |  |  |
| Education level, n (%) |  |  |  |
| < High school | 6563 (8.3) | 4200 (6.6) | 2363 (15.3) |
| High school | 22516 (28.4) | 16886 (26.5) | 5630 (36.5) |
| University/college ≤ 4 years | 31908 (40.3) | 26954 (42.3) | 4954 (32.2) |
| University/college > 4 years | 18216 (23.0) | 15752 (24.7) | 2464 (16.0) |
| Total | 79203 | 63792 | 15411 |
| Missing | 1746 (2.2) | 1366 (2.1) | 380 (2.4) |
| Diagnosis of endometriosis^a^,  n (%) |  |  |  |
| Yes | 944 (1.2) | 732 (1.1) | 212 (1.3) |
| No | 80005 (98.8) | 64426 (98.9) | 15579 (98.7) |
| Total | 80949 | 65158 | 15791 |
| Missing | 0 |  |  |
| Menstrual cycle regularity^b^,  n (%) |  |  |  |
| Regular | 59900 (74.2) | 49001 (75.4) | 4843 (69.2) |
| Irregular | 20829 (25.8) | 15986 (24.6) | 10899 (30.8) |
| Total | 80729 | 64987 | 15742 |
| Missing | 220 (0.3) | 171 (0.3) | 49 (0.3) |
| Body mass index, n (%) |  |  |  |
| <18.5 | 2554 (3.3) | 1846 (2.9) | 708 (4.7) |
| 18.5-24.9 | 51448 (65.5) | 41591 (65.7) | 9857 (64.8) |
| 25.0-29.9 | 17047 (21.7) | 13981 (22.1) | 3066 (20.2) |
| ≥30.0 | 7458 (9.5) | 5875 (9.3) | 1583 (10.4) |
| Total | 78507 | 63293 | 15214 |
| Missing | 2442 (3.0) | 1865 (2.9) | 577 (3.7) |
| Mean | 24.0 | 24.0 | 23.9 |

^a^Self-reported. ^b^Regular defined as menstruation at regular intervals of 21-35 days.
