# Supplementary material for: Sexual intercourse frequency and fecundability in the Norwegian mother, father and child cohort study
Source: PLoS One. 2026 Sep 23;21(9):e0357286. doi: 10.1371/journal.pone.0357286 (PMC13600493; doi:10.1371/journal.pone.0357286)
Supplement: Supplementary file 2 [file pone.0357286.s002.docx]

**S2 Table. Time to pregnancy by categories of intercourse frequency.**

| Time to pregnancy, n (%) | Total | <1 | 1-2 | 3-4 | >4 |
| --- | --- | --- | --- | --- | --- |
| ≤6 months | 52332 (81.5) | 7166 (79.3) | 18336 (80.5) | 19522 (81.6) | 7308 (86.5) |
| ≤12 months | 59605 (92.9) | 8285 (91.7) | 21047 (92.3) | 22223 (92.9) | 8050 (95.3) |
| >12 months | 4588 (7.2) | 752 (8.3) | 1745 (7.7) | 1690 (7.1) | 401 (4.8) |
| Total | 64193 | 9037 | 22792 | 23913 | 8451 |
| Missing | 0 | 0 | 0 | 0 | 0 |
| Mean (95% CI) | 4.5 | 4.8 (4.7-4.9) | 4.7 (4.6-4.7) | 4.5 (4.4-4.5) | 3.7 (3.6-3.8) |
