# Supplementary material for: Sexual intercourse frequency and fecundability in the Norwegian mother, father and child cohort study
Source: PLoS One. 2026 Sep 23;21(9):e0357286. doi: 10.1371/journal.pone.0357286 (PMC13600493; doi:10.1371/journal.pone.0357286)
Supplement: Supplementary file 3 [file pone.0357286.s003.docx]

**S3 Table. Fecundability ratio (FR) by categories of intercourse frequency stratified by parity.**

| Parity | Intercourse frequency  (times per week) | Participants, n (%) | FR, crude (95% CI) | FR, adjusted^a^ (95% CI) |
| --- | --- | --- | --- | --- |
| 0 | >4 | 5284 (15.6) | 1.27 (1.23-1.30) | 1.23 (1.20-1.27) |
|  | 3-4 | 13815 (40.7) | 1.05 (1.03-1.07) | 1.03 (1.01-1.05) |
|  | 1-2 | 11270 (33.2) | 1 (reference) | 1 (reference) |
|  | <1 | 3595 (10.6) | 0.95 (0.91-0.98) | 0.97 (0.93-1.00) |
|  |  |  |  |  |
| 1 | >4 | 2036 (9.9) | 1.13 (1.09-1.18) | 1.10 (1.05-1.14) |
|  | 3-4 | 6712 (32.8) | 1.00 (0.98-1.03) | 0.98 (0.95-1.01) |
|  | 1-2 | 7829 (38.2) | 1 (reference) | 1 (reference) |
|  | <1 | 3918 (19.1) | 1.00 (0.96-1.03) | 1.02 (0.99-1.06) |
|  |  |  |  |  |
| ≥2 | >4 | 1131 (11.6) | 1.06 (1.00-1.12) | 1.10 (1.04-1.17) |
|  | 3-4 | 3386 (34.8) | 1.04 (1.00-1.08) | 1.03 (0.99-1.07) |
|  | 1-2 | 3693 (37.9) | 1 (reference) | 1 (reference) |
|  | <1 | 1524 (15.7) | 1.03 (0.98-1.09) | 1.05 (0.99-1.11) |

^a^Adjusted for age and education level
