# Supplementary material for: Sexual intercourse frequency and fecundability in the Norwegian mother, father and child cohort study
Source: PLoS One. 2026 Sep 23;21(9):e0357286. doi: 10.1371/journal.pone.0357286 (PMC13600493; doi:10.1371/journal.pone.0357286)
Supplement: Supplementary file 4 [file pone.0357286.s004.docx]

**S4 Table. Fecundability ratio (FR) by categories of intercourse frequency stratified by body mass index (BMI) group.**

| BMI | Intercourse frequency  (times per week) | Participants, n (%) | FR, crude (95% CI) | FR, adjusted^a^ (95% CI) |
| --- | --- | --- | --- | --- |
| <18.5 | >4 | 283 (16.0) | 1.18 (1.05-1.34) | 1.18 (1.04-1.34) |
|  | 3-4 | 649 (36.7) | 1.07 (0.97-1.18) | 1.06 (0.96-1.17) |
|  | 1-2 | 606 (34.3) | 1 (reference) | 1 (reference) |
|  | <1 | 230 (13.0) | 1.01 (0.88-1.16) | 1.03 (0.90-1.19) |
|  |  |  |  |  |
| 18.5-24.9 | >4 | 5268 (13.1) | 1.19 (1.16-1.22) | 1.16 (1.13-1.19) |
|  | 3-4 | 15326 (38.1) | 1.02 (1.00-1.04) | 1.01 (0.99-1.03) |
|  | 1-2 | 14156 (35.2) | 1 (reference) | 1 (reference) |
|  | <1 | 5510 (13.7) | 1.00 (0.97-1.02) | 1.02 (0.99-1.05) |
|  |  |  |  |  |
| 25.0-29.9 | >4 | 1710 (12.7) | 1.19 (1.14-1.25) | 1.17 (1.12-1.23) |
|  | 3-4 | 4800 (35.7) | 1.00 (0.97-1.04) | 0.99 (0.96-1.03) |
|  | 1-2 | 4963 (36.9) | 1 (reference) | 1 (reference) |
|  | <1 | 1980 (14.7) | 1.01 (0.96-1.05) | 1.02 (0.97-1.07) |
|  |  |  |  |  |
| ≥30.0 | >4 | 776 (13.9) | 1.16 (1.08-1.25) | 1.15 (1.06-1.24) |
|  | 3-4 | 2001 (35.8) | 1.05 (0.99-1.16) | 1.05 (0.99-1.11) |
|  | 1-2 | 1991 (35.6) | 1 (reference) | 1 (reference) |
|  | <1 | 826 (14.8) | 0.97 (0.90-1.05) | 0.98 (0.91-1.07) |

^a^Adjusted for age and education level
