# Supplementary material for: Sexual intercourse frequency and fecundability in the Norwegian mother, father and child cohort study
Source: PLoS One. 2026 Sep 23;21(9):e0357286. doi: 10.1371/journal.pone.0357286 (PMC13600493; doi:10.1371/journal.pone.0357286)
Supplement: Supplementary file 5 [file pone.0357286.s005.docx]

**S5 Table. Fecundability ratio (FR) by categories of intercourse frequency excluding women with irregular menstrual cycles^a^ (N=15719).**

| Intercourse frequency  (times per week) | Participants, n (%) | FR, crude (95% CI) | FR, adjusted ^b^ (95% CI) |
| --- | --- | --- | --- |
| >4 | 6153 (13.0) | 1.20 (1.18-1.23) | 1.17 (1.14-1.20) |
| 3-4 | 17605 (37.1) | 1.04 (1.03-1.06) | 1.03 (1.01-1.04) |
| 1-2 | 16974 (35.7) | 1 (reference) | 1 (reference) |
| <1 | 6756 (14.2) | 0.97 (0.95-1.00) | 1.00 (0.97-1.02) |
| Total | 47488 |  |  |

^a^Defined as not menstruation at regular intervals of 21-35 days. ^b^Adjusted for age and education level.
