# Supplementary material for: Sexual intercourse frequency and fecundability in the Norwegian mother, father and child cohort study
Source: PLoS One. 2026 Sep 23;21(9):e0357286. doi: 10.1371/journal.pone.0357286 (PMC13600493; doi:10.1371/journal.pone.0357286)
Supplement: Supplementary file 6 [file pone.0357286.s006.docx]

**S6 Table. Fecundability ratio (FR) by categories of intercourse frequency, excluding women with endometriosis**^a^ **(N=678).**

| Intercourse frequency  (times per week) | Participants, n (%) | FR, crude (95% CI) | FR, adjusted^b^ (95% CI) |
| --- | --- | --- | --- |
| >4 | 8370 (13.2) | 1.18 (1.15-1.20) | 1.16 (1.13-1.18) |
| 3-4 | 23667 (37.3) | 1.02 (1.01-1.04) | 1.01 (0.99-1.02) |
| 1-2 | 22544 (35.5) | 1 (reference) | 1 (reference) |
| <1 | 8934 (14.1) | 0.99 (0.97-1.02) | 1.01 (0.99-1.03) |
| Total | 63515 |  |  |

^a^ Self-reported. ^b^ Adjusted for age and education level.
