# Supplementary material for: Sexual intercourse frequency and fecundability in the Norwegian mother, father and child cohort study
Source: PLoS One. 2026 Sep 23;21(9):e0357286. doi: 10.1371/journal.pone.0357286 (PMC13600493; doi:10.1371/journal.pone.0357286)
Supplement: Supplementary file 7 [file pone.0357286.s007.docx]

**S7 Table. Fecundability ratio (FR) by categories of intercourse frequency with alternative adjustment model^a^**.

| Intercourse frequency  (times per week) | Participants, n (%) | FR, crude (95% CI) | FR, adjusted^a^ (95% CI) |
| --- | --- | --- | --- |
| >4 | 8074 (13.1) | 1.18 (1.16-1.21) | 1.18 (1.15-1.20) |
| 3-4 | 22960 (37.3) | 1.02 (1.01-1.04) | 1.02 (1.00-1.04) |
| 1-2 | 21871 (35.6) | 1 (reference) | 1 (reference) |
| <1 | 8599 (14.0) | 1.00 (0.97-1.02) | 1.00 (0.98-1.03) |
| Total | 61504 |  |  |

^a^Adjusted for age, education level, BMI, parity, menstrual cycle regularity.
